# Liver immunopathogenesis in fatal cases of dengue in children: detection of viral antigen, cytokine profile and inflammatory mediators

**DOI:** 10.3389/fimmu.2023.1215730

**Published:** 2023-06-30

**Authors:** Leandro Junqueira Moragas, Felipe de Andrade Vieira Alves, Lucca de Lima Siqueira Oliveira, Natália Gedeão Salomão, Caio Gonçalves Azevedo, Jemima Fuentes Ribeiro da Silva, Carlos Alberto Basílio-de-Oliveira, Rodrigo Basílio-de-Oliveira, Ronaldo Mohana-Borges, Jorge José de Carvalho, Fernando Colonna Rosman, Marciano Viana Paes, Kíssila Rabelo

**Affiliations:** ^1^Laboratório Interdisciplinar de Pesquisas Médicas, Instituto Oswaldo Cruz, Fundação Oswaldo Cruz, Rio de Janeiro, Brazil; ^2^Laboratório de Ultraestrutura e Biologia Tecidual, Universidade do Estado do Rio de Janeiro, Rio de Janeiro, Brazil; ^3^Anatomia Patológica, Universidade Federal do Estado do Rio de Janeiro, Rio de Janeiro, Brazil; ^4^Laboratório de Genômica Estrutural, Instituto de Biofísica Carlos Chagas Filho, Universidade Federal do Rio de Janeiro, Rio de Janeiro, Brazil; ^5^Serviço de Anatomia Patológica, Hospital Clementino Fraga Filho, Universidade Federal do Rio de Janeiro, Rio de Janeiro, Brazil

**Keywords:** hepatic damage, dengue virus, histopathology, inflammation, children

## Abstract

**Introduction:**

Dengue virus (DENV), the etiologic agent of dengue fever illness, represents a global public health concern, mainly in tropical and subtropical areas across the globe. It is well known that this acute viral disease can progress to severe hemorrhagic stages in some individuals, however, the immunopathogenic basis of the development of more severe forms by these patients is yet to be fully understood.

**Objective:**

In this context, we investigated and characterized the histopathological features as well as the cytokine profile and cell subpopulations present in liver tissues from three fatal cases of DENV in children.

**Methods:**

Hematoxylin and Eosin, Periodic Acid Schiff and Picro Sirius Red staining were utilized for the histopathological analysis. Immunohistochemistry assay was performed to characterize the inflammatory response and cell expression patterns.

**Results:**

Vascular dysfunctions such as hemorrhage, vascular congestion and edema associated with a mononuclear infiltrate were observedin all three cases. Liver tissues exhibited increased presence of CD68+ and TCD8+ cells as well as high expression of MMP-9, TNF-a, RANTES, VEGFR-2 mediators. Viral replication was confirmed by the detection of NS3 protein.

**Conclusion:**

Taken together, these results evidenced key factors that may be involved in the development of severe alterations in liver tissues of children in response to DENV infection.

## 1 Introduction

Dengue fever (DF) is considered a major public health concern due to its fast worldwide spread and high incidence rate in tropical and subtropical countries around the globe (1, 2). The etiologic agent, dengue virus (DENV), is mainly transmitted by the bite of the *Aedes aegypti* and *Aedes albopictus* mosquitoes (3). According to the World Health Organization (WHO), the incidence of the disease has increased 30 times around the world in the last 50 years, with an estimated total of 50 million infections per year (4, 5).

In most infected individuals, DF is limited to a mild form illness, requiring minimal supportive treatment, however the disease may progress to more severe forms, characterized by alterations in hemostasis, increase in vascular permeability and plasma leakage (6–8). Nowadays, classification, management and control of dengue are standardized by the 2009 guideline of World Health Organization, which classifies the disease in two categories: non-severe dengue and severe dengue (4). Most deaths are associated with severe dengue form, which can cause dysfunction and inflammation in several different organs that, concomitantly with absence of supportive care, can increase the mortality rate to 4% of cases (9–11).

Although several organs can be affected, hepatic involvement is recognized as a common complication in dengue infection (12, 13). The correlation between dengue virus infection and hepatic injury has already been demonstrated both in adults (14–17) and children (18, 19). Also, different studies support the interconnection between increased serum levels of aspartate aminotransferase (AST) and alanine aminotransferase (ALT) and liver involvement in the acute phase of the disease (14, 20, 21). Therefore, it is possible that the virus can lead to acute liver failure by either direct viral toxicity to hepatocytes or dysregulated immunologic response to the virus (22).

Previous reports of our group revealed severe morphological alterations such as hemorrhage, edema, steatosis, focal areas of necrosis and large amounts of inflammatory infiltrates in the liver of DENV-infected adults (23). The presence of DENV antigens was also confirmed by immunohistochemistry, as well as characterization of CD8^+^T, CD4^+^T and CD68^+^ cells and cytokines/chemokines such as IFN-γ and TNF-α (24). More recently, we also observed renal tissues of dengue fatal cases in children associated to DENV with the development of acute renal injury and glomerulonephritis (25). Other studies approaching dengue fatal cases evidenced alterations in histopathology and cytokines profile of other organs such as spleen, lung, heart, kidney, pancreas and placenta (23, 24, 26–30).

In this context, the present work aimed to investigate the histopathological and ultrastructural aspects of liver tissues from three fatal cases of dengue in children that occurred between the period of 2008 to 2012, as well as address the inflammatory immune profile. These patients developed severe dengue that seriously affected other organs besides the liver. Therefore, the analysis of liver biochemical parameters and the associated damages in the parenchyma may help to elucidate the immunological mechanisms involved in the pathogenesis of dengue in hepatic tissues and how it contributed to the final outcome of these patients.

## 2 Materials and methods

### 2.1 Ethical considerations

All procedures performed during this study were approved by the Ethics Committee of the Oswaldo Cruz Foundation/FIOCRUZ (CAEE: 47525115.3.0000.5248).

### 2.2 Clinical history of patients

Between 2008 and 2012, our group obtained samples of liver tissues from three children after their deaths due to DENV infection during Rio de Janeiro outbreaks. All patients had the diagnosis of DF confirmed by the presence of anti-DENV IgM antibodies or detection of the NS1 antigen by immunochromatography.

#### 2.2.1 Controls

The negative controls of liver samples were obtained from three fatalities ranging from 8 to 13 years old presenting no signs of infectious diseases or hepatic disorder.

##### 2.2.1.1 Case 1

Male patient, melanodermic, 7-years-old, born in Rio de Janeiro, Brazil, admitted at the Hospital Municipal Jesus with fever, nausea, hematemesis, hypohydration and bleeding gums on January 30, 2008. On January 29, 2008, after 5 days with high fever, the mother sought medical care at a health center, where the child was treated with amoxicillin for the diagnosis of “infection”. The individual’s physical condition worsened, which made him return to the medical care center the next day, where the presumptive diagnosis of dengue was made by confirmatory serology for anti-DENV IgM antibodies and hospitalization was provided. At the physical examination, the patient revealed pulmonary rales during auscultation, severe pain in the abdomen with mild hepatomegaly and large pleural effusion. After two days of hospitalization, blood culture was positive for bacteria *Pseudomonas aeruginosa*, probably acquired at the hospital premises. In the following days, the clinical course evolved to refractory shock, renal failure and acute respiratory distress syndrome (ARDS). On the fourteenth day of hospitalization, February 12, 2008, the patient presented anuria, hypothermia, bradycardia, absence of arterial pulse and cardiopulmonary arrest followed by death with clinical diagnosis of hemorrhagic dengue. The last blood count revealed leukocytosis, neutrophilia, lymphopenia, hyperchromic microcytic anemia and thrombocytopenia. Necropsy data of the liver showed acute hepatitis with steatosis and foci of necrosis and centrilobular hemorrhage.

##### 2.2.1.2 Case 2

Female patient, with 9 years-old, born in Rio de Janeiro, Brazil, admitted at the Hospital Municipal Jesus in 2011, had fever and general malaise on April 23, 2011, being taken to the medical care center on April 24, 2011. The physical examination showed severe prostration, drowsiness, dehydration, tachycardia, globular abdomen with painful hepatomegaly and petechiae on the face and lower limbs. She was treated with ranitidine, dipyrone, and required replacement and maintenance hydration with saline and glucose solutions with addition of sodium chloride and potassium chloride. On April 25, 2011, she was transferred to the intensive care unit, where blood count performed revealed leukopenia, neutropenia, lymphopenia, anemia and thrombocytopenia. In the same day of hospitalization, the patient received hydro electrolytic replacement and hemodynamic control, with usual medication, however, the clinical condition worsened, evolving to bradycardia followed by cardiopulmonary arrest without response to medication and external cardiac massage, culminating in her death. The main diagnosis was dengue confirmed by immunochromatographic test with detection of the NS1 antigen of DENV; and the cause of death was hemorrhagic shock, with multiple organ failure syndrome. Necropsy data revealed hepatomegaly, marked congestion, hemorrhage and necrosis of hepatic lobules secondary to acute ischemia.

##### 2.2.1.3 Case 3

Male patient, with 10 years old, born in Rio de Janeiro, Brazil, admitted at the Hospital Municipal Jesus in 2012, presented fever, emesis, headache and prostration at night on April 14, 2012. On April 18, 2012, the mother seeked medical attention in the Emergency Care Unit, where he received initial medical treatment and then, referred to the Dengue Center. On the same day, blood counts performed showed leukopenia and thrombocytopenia, which indicated a possible case of dengue. Later on, the patient was taken to hospitalization at the intensive care unit, where physical examination revealed drowsiness, globular abdomen, edema, painful hepatomegaly and exanthema. Hydrothorax and ascites were observed through imaging exams. On April 19, 2012, he was cyanotic, with dyspnea, and orotracheal intubation was fulfilled. Dengue diagnosis was confirmed by serology using Dengue IgM kit—Elisa Capture (PanBio) on the day of intubation. The clinical course evolved to shock with absence of central and peripheral arterial pulse, culminating in cardiac arrest having his death recorded on April 20, 2011. The main diagnosis was hemorrhagic dengue; the cause of death was brain edema. Necropsy data revealed steatosis, hemorrhage and necrosis of hepatic lobules, associated with sinusoidal congestion secondary to acute ischemia.

The condition of each case is summarized in **Table 1** and all clinical data of the three cases analyzed in this study are available in **Supplementary Material 1**.

**Table 1:** Clinical data and necropsy records from the three dengue fatal cases.

Dengue cases	Case 1	Case 2	Case 3
**Age**	7 years and 8 months	9 years and 11 months	10 years and 8 months
**Sex**	Male	Female	Male
**Symptoms**	Fever Nausea Emesis Abdominal pain Gingival bleeding Hematemesis	Fever Prostration Emesis Drowsiness Abdominal pain Malaise	Fever Prostration Emesis Drowsiness Abdominal pain Rash
**Laboratory findings**	Leukopenia Lymphopenia Thrombocytopenia	Leukopenia Neutropenia Lymphopenia Anemia Thrombocytopenia	Leukopenia Thrombocytopenia
**Physical examination**	Painful abdomen Pleural effusion Low-amplitude arterial pulses Blood pressure of 80x50 mmHg	Painful abdomen Hepatomegaly Blood pressure of 90x60mmHg Petechiae	Painful globular abdomen Blood pressure 110x62mmHg Tach dyspneic
**Necropsy data**	Hepatomegaly Acute hepatitis with steatosis Foci of necrosis and centrilobular hemorrhage. Intrahepatic cholestasis.	Hepatomegaly Hepatic congestion Hemorrhage and necrosis in zones 2 and 3 of hepatic lobules	Hepatic steatosis Hepatic congestion Hemorrhage and necrosis in zones 3 (perivenular) and 2 (mediozonal) of the hepatic lobules
**Hospitalization period**	14 days	1 day	2 days
**Confirmed diagnosis**	IgM positive	NS1 positive	IgM positive
**Classification**	Severe dengue	Severe dengue	Severe dengue

### 2.3 Histopathological analysis

The liver tissues fragments from necropsies were fixed in 10% formaldehyde, pH = 7.2, and processed and blocked-in paraffin resin. Sections of 5 μm thickness were made in a microtome (American Optical, Spencer model), and mounted in glass slides. Before staining, the slides were deparaffinized in three baths of xylene and rehydrated with decreasing concentrations of ethanol (100 to 70%). Then, sections were submitted to standard and special staining with Hematoxylin and Eosin (H.E.), Periodic Acid Schiff (PAS), Picro Sirius Red and prepared for visualization under a Nikon ECLIPSE E600 microscope. Photomicrographs were captured using Image-Pro Plus software version 7 (Media Cybernetics).

### 2.4 Immunohistochemistry procedures

For detection of NS3 protein as well as characterization of cell populations, cytokines and inflammatory mediators by immunohistochemistry, sections were treated as described in a previous work (31). Sections were then incubated overnight at 4˚C with the following primary antibodies: anti-NS3 (produced in house, expressed in *Escherichia coli*, purified and inoculated in BALB/c mice; dilution 1:100), rabbit anti-human CD4^+^ monoclonal antibody clone SP35 (Spring Bioscience, CA, USA; dilution 1:100), mouse anti-human CD8^+^ monoclonal antibody clone C8/144B (Dako, CA, USA; dilution 1:200), mouse anti-human CD68^+^ monoclonal antibody clone EBM11 (Dako, CA, USA; dilution 1:200), rabbit anti-human TNF-α polyclonal antibody clone ab6671 (Abcam, MA, USA; dilution 1:200), rabbit anti-human RANTES monoclonal antibody clone ab189841 (Abcam, MA, USA; dilution 1:200), rabbit anti-human VEGFR-2 monoclonal antibody clone SP123 (Spring Bioscience, CA, USA; dilution 1:100), rabbit anti-human VCAM-1 monoclonal antibody clone ab134047 (Abcam, MA, USA; dilution 1:100), mouse anti-human MMP-9 monoclonal antibody clone sc-21733 (Santa Cruz Biotechnology, TX, USA; dilution 1:100). On the second day, sections were washed three times and incubated with secondary antibody (REVEAL complement, Spring Bioscience, CA, USA) for 10 min and with rabbit anti-mouse IgG-HRP conjugate (REVEAL polyvalent HRP, Spring Bioscience, CA, USA) for 15 min at room temperature; followed by reveal of the reaction with the substrate for peroxidase diaminobenzidine (Dako, CA, USA). Counterstaining was performed with Harry’s hematoxylin (Sigma, MO, USA) and then, sections were prepared for visualization under a Nikon ECLIPSE E600 microscope.

### 2.5 Liver tissue quantifications and morphometry

For each specific antibody reaction, 20 fields were randomly acquired at 1000x magnification using the software Image-Pro Plus version 7 (Media Cybernetics, MD, USA) from samples of both control and infected liver tissues. The positive cells within each field were quantified and the mean number of positive cells per field was determined. For the semiquantitative analysis of the tissue damages, an arbitrary scale of 0–4 (0 = none; 1 = mild; 2 = moderate; 3 = severe; 4 = very severe) was implemented according to six criteria observed in each quadrant: tissue necrosis; presence of inflammatory infiltrate; vascular congestion; hemorrhage; edema; steatosis. All photomicrographs obtained were realized by an individual blinded to the diagnosis associated with the tissue sample. Figures present representative fields to best convey the quantification results.

### 2.6 Statistical analysis

Data were analyzed with GraphPad Prism software version 6.0 (La Jolla, CA, USA) using non-parametric statistical tests. Significant statistical differences between the analyzed groups (controls and cases) were determined using Mann-Whitney test with a threshold of p < 0.05.

## 3 Results

### 3.1 Serum levels of aspartate aminotransferase and alanine aminotransferase in dengue-patients during hospitalization days

**Figure 1:**
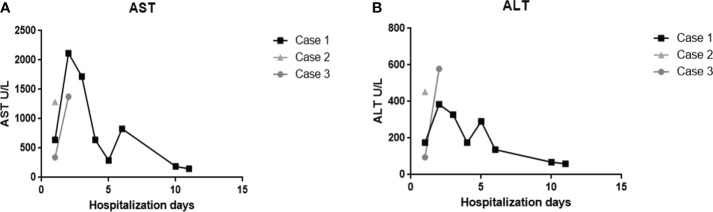
Evaluation of AST and ALT enzymes of the three fatal cases of DENV-infected infant patients. Serum levels of **(A)** aspartate aminotransferase and **(B)** alanine aminotransferase during hospitalization days.

Levels of aspartate aminotransferase (AST) and alanine aminotransferase (ALT) were measured during the hospitalization period. All three cases presented serum levels above the reference values for both enzymes (**Figures 1A, B**). The first case showed the highest levels of AST in the second day of hospitalization (**Figure 1A**), which reached its maximum peak of 2.117 U/L, an increase of approximately 64 times in relation to the maximum limit (reference value: 0 – 32 U/L). The highest levels of ALT were observed in the third case (**Figure 1B**), which reached its maximum peak of 579 U/L at the second day of hospitalization, corresponding to an increase of 10 times of the maximum limit (reference values: 0 – 31 U/L).

### 3.2 Histopathological analysis

**Figure 2:**
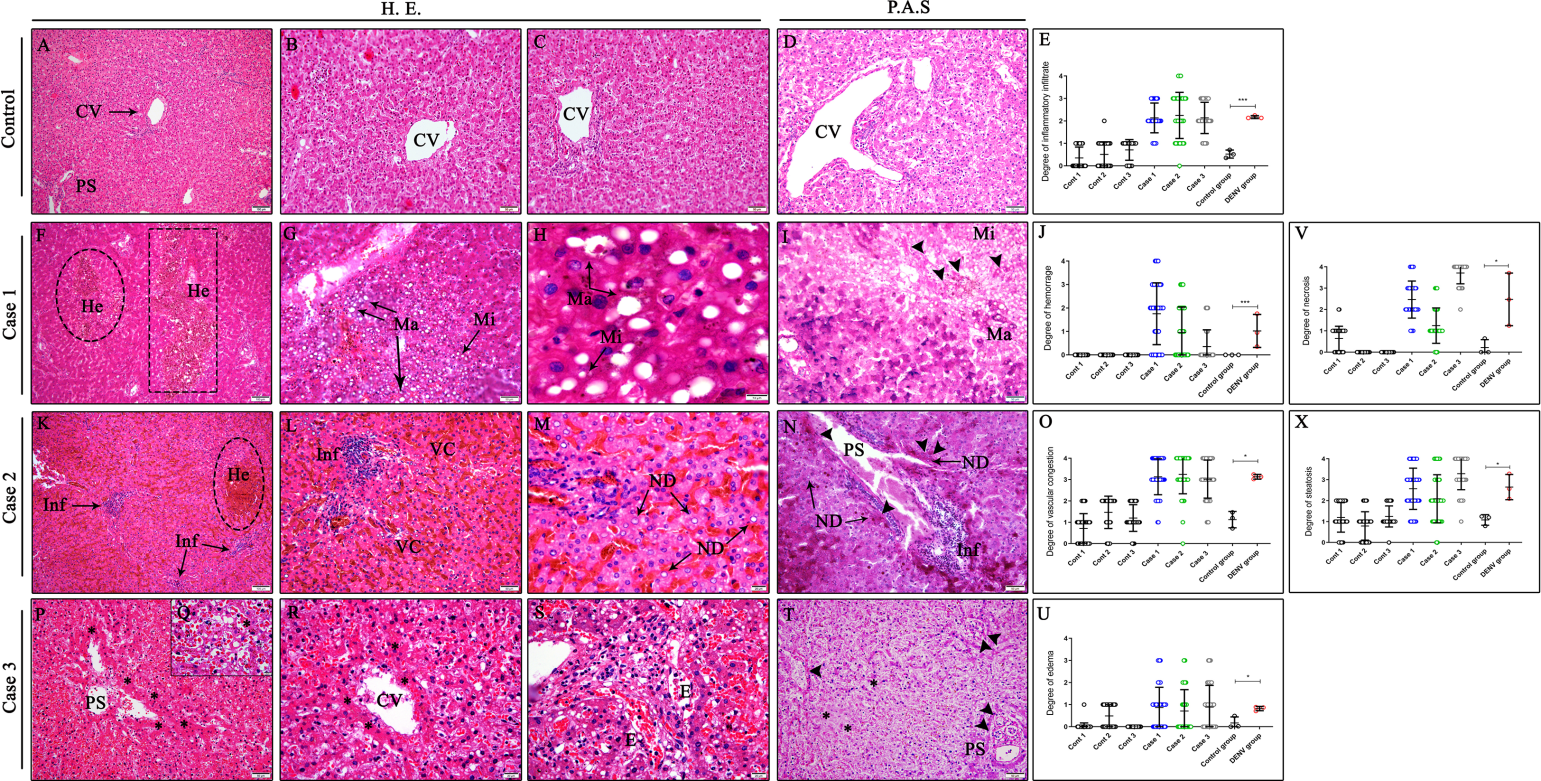
Histopathological aspects of liver tissues from DENV fatal cases of children. **(A–D)** Liver of non-dengue cases stained with H.E. and PAS presenting well-preserved hepatocytes and normal aspects. Liver sections of dengue fatal cases presenting several hepatic injuries, such as **(G, H)** macro and microsteatosis, **(P–R)** necrosis, **(M)** nuclear degeneration and **(F, L, S)** circulatory features including hemorrhage, vascular congestion and edema associated with an **(K)** inflammatory infiltrate. **(I, N, T)** PAS special stain revealing deposits of glycogen in hepatocytes within steatosis and portal space areas in the three dengue fatal cases. (CV) Centrilobular vein; (PS) Portal space; (He) Hemorrhage; (Ma) Macrosteatosis; (Mi) Microsteatosis; (Inf) Inflammatory infiltrate; (VC) Vascular congestion; (ND) Nuclear degeneration; **(E)** Edema; (Asterisk) Necrosis areas; (Arrow head) PAS positive stain. **(E, J, O, U, V, X)** Semiquantitative analysis of alterations in the liver tissue of infected children and control. Asterisks indicate significant differences using F statistical test (*p < 0.05 and ***p < 0.0001). **(A, F, K)** Magnification 10x, **(B–D, G, I, L, N, P, T)** magnification 20x, **(H, M, Q, R, S)** magnification 100x.

Evaluation of the liver tissue samples showed circulatory damages and necrosis of hepatocytes in all lobules of DENV studied cases. Several lesions were observed in dengue cases, including steatosis, which was characterized by an abnormal retention of lipids around the nucleus of hepatocytes presented as either large (macrosteatosis) or small fat vacuoles (microsteatosis) (**Figures 2G, H**). Vascular dysfunction was characterized by extensive areas of hemorrhage, edema and vascular congestion observed in all three fatal cases (**Figures 2F, L, S**). Regarding circulatory changes, an intense mononuclear inflammatory infiltrate was also observed in case 2 (**Figure 2K**). Additionally, the hepatic parenchyma of dengue cases presented focal areas of necrosis especially around the centrilobular vein and portal space (**Figures 2P–R**). Cell death process was also evidenced by nuclear degeneration findings (**Figure 2M**). As expected, the liver of non-dengue patients showed absence of changes in the parenchyma, with hepatocytes presenting regular morphology and well-preserved centrilobular veins as well as sinusoid capillaries (**Figures 2A–D**). Periodic Acid Schiff, a special stain that evidences glycogen and glycoproteins, was performed to observe deposits of these molecules in liver tissues. Positive areas for PAS stain, characterized by a purple color, were observed in hepatocytes in the parenchyma with steatosis (**Figure 2I**) including extensive areas of nuclear degeneration associated with inflammatory infiltrates around portal space (**Figures 2N, T**). Semiquantitative analysis of damages was carried out according to six different parameters: presence of inflammatory infiltrate, hemorrhage, vascular congestion, edema, steatosis and necrosis. Five distinct degrees (0= none; 1 = low; 2 = moderate; 3 = severe; 4 = very severe) were stipulated for each field photographed according to the level of damage observed in each quadrant. The criteria for presence of inflammatory infiltrate and hemorrhage had a low to moderate intensity in most of the cases fields, however, in control tissues, these criteria were barely seen (**Figures 2E, J**). On the other hand, the criteria of vascular congestion, steatosis and necrosis had a severe involvement (3/4 quadrants of the field) in most of the cases fields (**Figures 2O, V, X**) while the parameter of edema had a low pattern in most fields acquired (**Figure 2U**).

### 3.3 Evaluation of fibrosis by collagen deposition and matrix metallopeptidase 9 expression and in liver tissues

**Figure 3:**
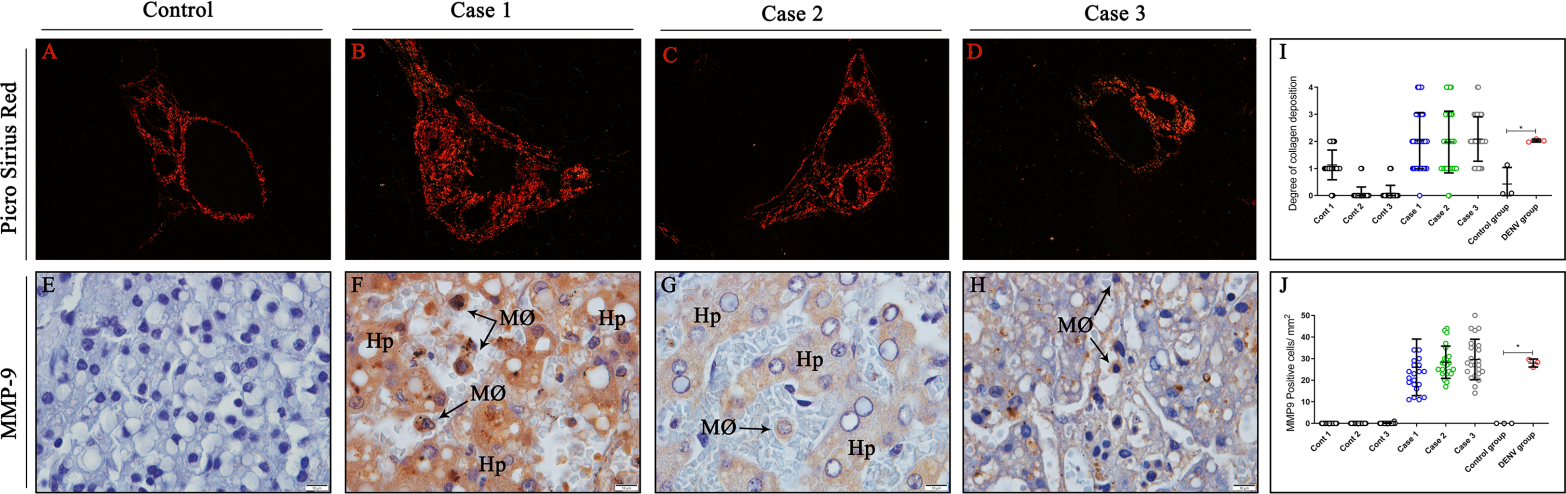
Analysis of hepatic fibrosis due to collagen deposition induced by MMP-9 expression. **(A)** Control liver tissues exhibiting regular distribution of collagen around portal space. Picro Sirius red stain evidencing collagen deposition around the portal space and midzonal regions of cases **(B)** 1 **(C)** 2 and **(D)** 3, respectively. **(E)** Control cases showing no expression of MMP-9. **(F–H)** Detection of MMP-9 in hepatocytes, macrophages and monocytes inside sinusoidal capillaries in dengue-liver tissues. **(I)** Semi-quantitative analysis of collagen deposition in all controls and cases. **(J)** Quantitative analysis of MMP-9 expression in liver tissues. (Hp) Hepatocytes; (MØ) Macrophages. Asterisks indicate significant differences using F statistical test (*p < 0.05). **(A–D)** Magnification 40x, **(E–H)** magnification 100x.

In order to analyze the degree of fibrosis in liver tissues, samples were stained with Picro Sirius Red, a special stain that evidences distinct types of collagens. As expected, control samples presented normal collagen deposition around portal space, which exhibited a regular thickness (**Figure 3A**). In contrast, liver dengue cases showed a moderate-to-intense deposition of type IV collagen around the portal space and in a few areas of the midzonal region of the hepatic parenchyma (**Figures 3B–D**). Quantification of the degree of collagen deposition revealed a significant increase in collagen deposition in the liver tissues from fatal cases compared to controls (**Figure 3I**). As metalloproteinases acts as key modulators of extracellular matrix, MMP-9, an enzyme that degrades type IV collagen, was also observed being expressed by hepatocytes, monocytes and macrophages in dengue-liver tissues (**Figures 3F–H**). Absence of MMP-9 expression was noted in control tissues (**Figure 3E**) as evidenced by quantification of this proteinase (**Figure 3J**).

### 3.4 Detection of viral antigen in hepatocytes and hyperplastic Kupffer cells with activated morphology in DENV-infected patients

**Figure 4:**
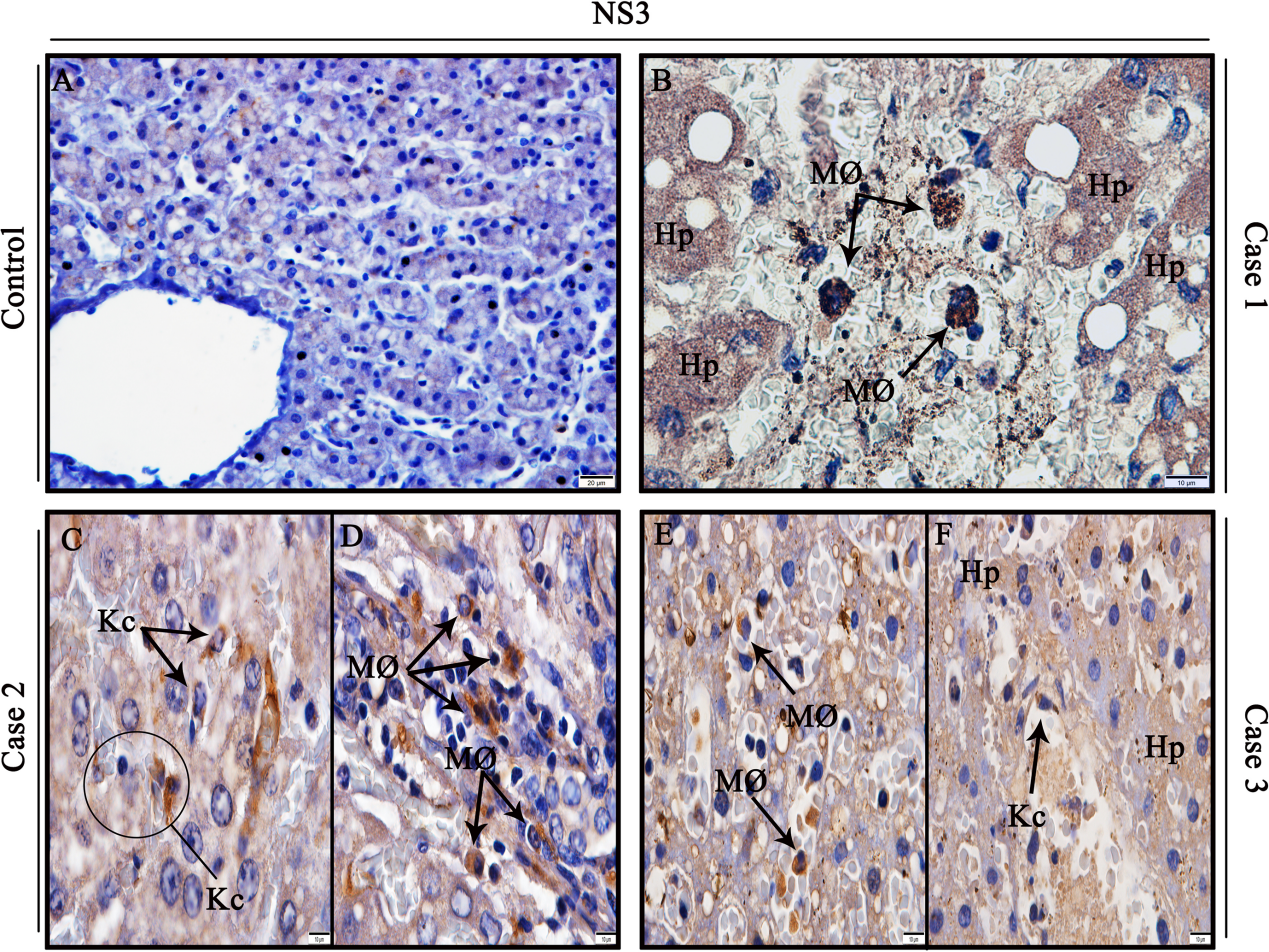
Detection of the NS3 protein antigen in liver tissues from DENV fatal cases of children. **(A)** Control liver tissues showing no presence of NS3 protein antigen. **(B)** Detection of NS3 antigen in hepatocytes and circulant macrophages in case 1. **(C, D)** Detection of NS3 antigen in Kupffer cells and macrophages in case 2. **(E, F)** Detection of NS3 antigen in macrophages, hepatocytes, and Kupffer cells in case 3. (Hp) Hepatocytes; (MØ) Macrophages; (Kc) Kupffer cells. **(A)** Magnification 40x, **(B–F)** magnification 100x.

To investigate sites of viral replication in the liver tissues of the studied cases, it was performed the detection of the dengue non-structural 3 (NS3) protein by immunohistochemistry assay. Positive reaction for NS3 antigen was indeed observed in all three dengue cases, mainly in circulant macrophages and hepatocytes in parenchyma (**Figures 4B, D, E**), as well as Kupffer cells (**Figures 4C, F**), evidencing viral replication in these cells. In contrast, non-dengue cases did not react with antibodies targeting NS3 antigen, revealing absence of viral replication in these tissues (**Figure 4A**).

### 3.5 Characterization of cell subpopulation patterns in infected liver tissues

**Figure 5:**
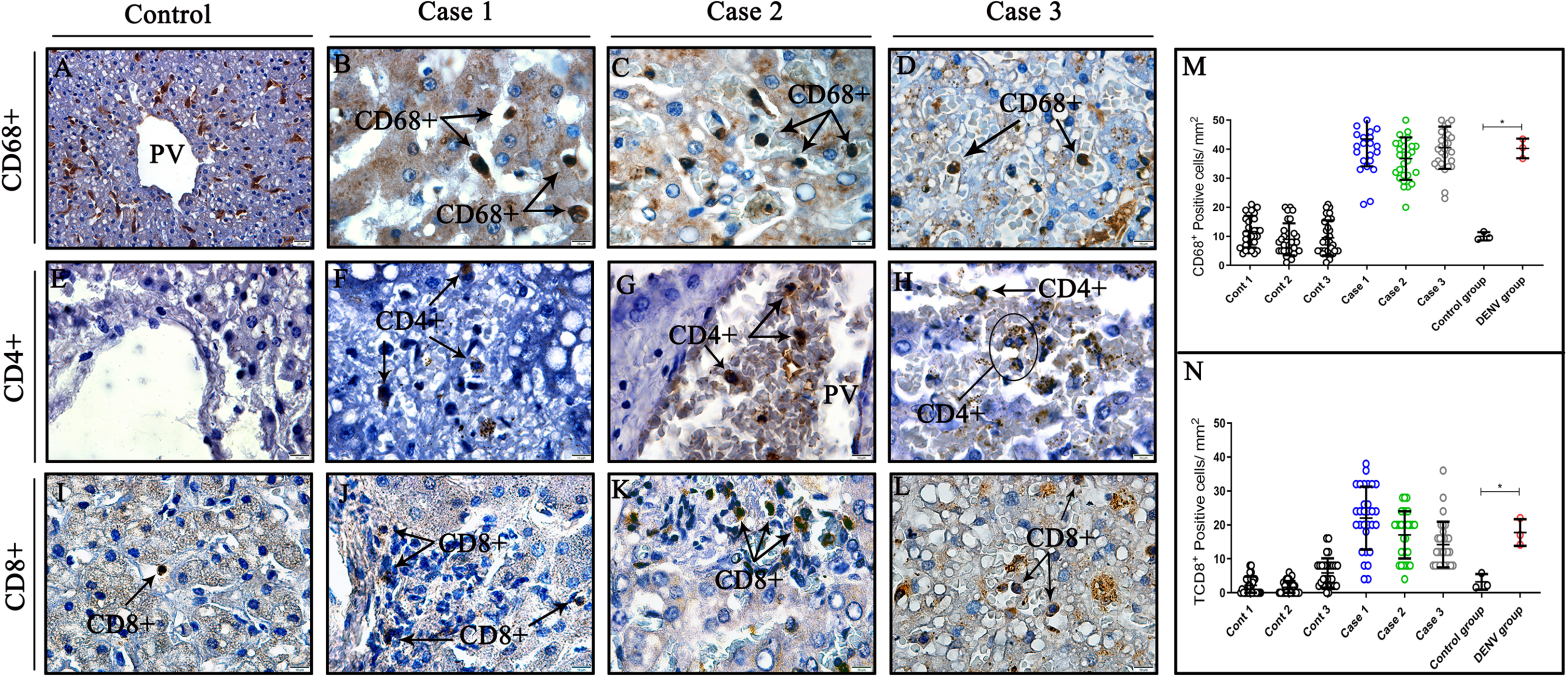
Characterization and quantification of cell subpopulation in liver tissues from DENV fatal cases of children. **(A, E, I)** Control tissues with little or no expression of CD68^+^, CD8^+^T CD4^+^T cells. **(B, C, D)** Liver tissues of dengue fatal cases expressing CD8^+^T cells in its parenchyma. **(F, G, H)** Few CD4^+^T lymphocytes detected around hepatocytes and vessels in liver infected tissues. **(J–L)** CD8^+^T cytotoxic lymphocytes detected in liver infected tissues. **(M)** Quantification of CD68^+^ cells. **(N)** Quantification of TCD8^+^ cells. (CD68^+^) Macrophages; (CD4^+^T) CD4^+^T lymphocytes; (CD8^+^T) CD8^+^T lymphocytes. **(A)** Magnification 40x, **(B–L)** magnification 100x. Asterisks indicate significant differences using F statistical test (*p < 0.05).

The mononuclear infiltrates observed in liver tissues of three fatal cases were composed by CD68^+^ cells (**Figures 5B–D**), which presented characteristics of hypertrophic morphology. The detection of CD8^+^ cells around hepatocytes also evidenced the presence of cytotoxic lymphocytes composing the inflammatory infiltrates (**Figures 5J–L**). Fewer CD4^+^ lymphocytes were detected in hepatic parenchyma (**Figure 5F**) and inside vessels (**Figures 5G, H**) of liver fatal cases, which did not enable its quantification. Control tissues also exhibited presence of CD68^+^ and CD8^+^ cells (**Figures 5A, I**), although, CD4^+^ cells were not observed in these cases (**Figure 5E**). Quantification of CD68^+^ cells revealed a significant increase in fatal cases compared to controls (**Figure 5M**) as well as CD8^+^ cells, which also indicated statistically significant increase between cases and controls (**Figure 5N**).

### 3.6 Characterization of cytokines and inflammatory mediators involved in vascular leakage in liver tissues

**Figure 6:**
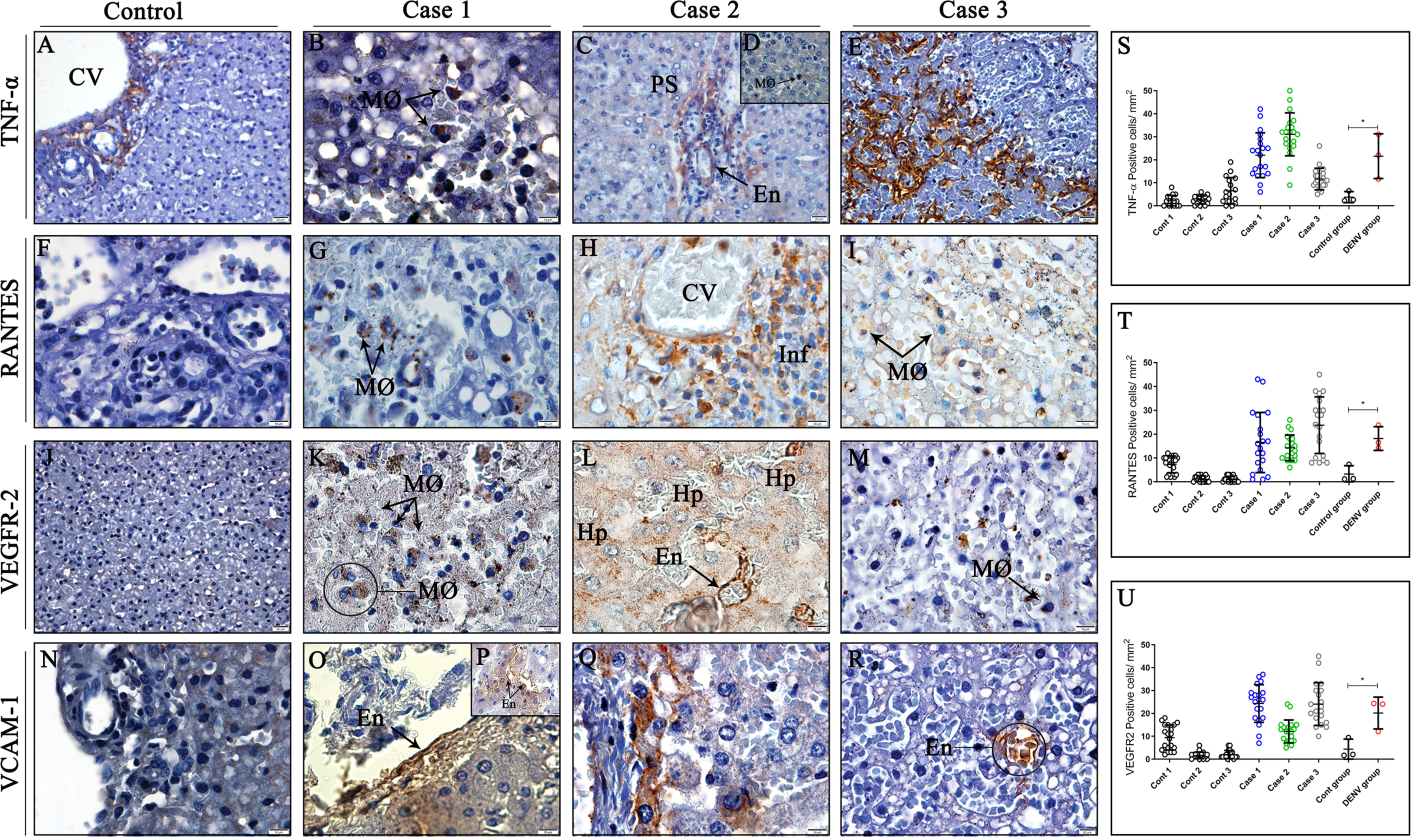
Detection and quantification of cytokines and inflammatory mediators in liver tissues from DENV fatal cases of children. **(A)** Control tissue presenting expression of TNF-α around the centrilobular vein. **(F, J, N)** Control tissues presenting little or none expression of RANTES, VEGFR-2 and VCAM-1. **(B, D)** Expression of TNF-α in macrophages in liver dengue cases. **(C, E)** TNF-α expression in endothelial cells of portal space and sinusoidal capillaries. **(G–I)** Expression of RANTES in macrophages and mononuclear infiltrates around centrilobular vein. **(K–M)** VEGFR-2 expression in hepatocytes, endothelial cells, and infiltrated macrophages. **(O, P, R)** Endothelial cells expressing VCAM-1 in their surface. **(Q)** Infiltrating cells perivascular exhibiting VCAM-1 expression. **(S–U)** Quantitative analysis of TNF-α, RANTES and VEGFR-2 expression in liver tissues. (CV) Centrilobular vein; (PS) Portal space; (Hp) Hepatocytes; (MØ) Macrophages; (Inf) Inflammatory infiltrate; (En) Endothelium. Asterisks indicate significant differences using F statistical test (*p < 0.05). **(A, C, E, J, R)** Magnification 40x, **(B, D, F–I, K-M, N-Q)** magnification 100x.

The inflammatory profile was primarily characterized by expression of TNF-α, RANTES, VEGFR-2 and VCAM-1, well-known markers implicated in DENV pathogenesis and progression of the disease. TNF-α expression was detected mainly in macrophages (**Figures 6B, D**) and endothelial cells of portal space and sinusoidal capillaries (**Figures 6C, E**), identified by their morphology. RANTES protein was found being expressed by macrophages and mononuclear infiltrates around centrilobular vein (**Figures 6G–I**), while VEGFR-2 was expressed by hepatocytes, endothelial cells and infiltrated macrophages in infected tissues (**Figures 6K–M**). VCAM-1, a protein that mediates adhesion of lymphocytes, macrophages and other cell types, was detected in the surface of endothelial cells (**Figures 6O, P, R**) and infiltrate cells perivascular (**Figure 6Q**). Non-dengue tissues exhibited little or none expression of RANTES, VEGFR-2 and VCAM-1 (**Figures 6F, J, N**), however, TNF-α was detected in cells around the centrilobular vein (**Figure 6A**). Quantitative analysis revealed significant increase in the expression of all three mediators in liver tissues from dengue-cases when compared to controls (**Figures 6S–U**).

## 4 Discussion

In general, the infection by DENV is usually self-limited to a mild illness with a fine prognosis (32). In spite of that, certain factors and conditions may predispose some individuals to develop the severe form of the disease, such as pre-existing comorbidities, history of previous infection of dengue, sex and age (33, 34). Children are considered a high-risk group for developing severe dengue, especially during a secondary infection with a heterologous DENV serotype (34, 35). The development of severe forms of the disease are associated with dysfunction in several organs due to inflammation, mainly the liver, leading to severe injuries in the organ and elevated serum hepatic enzymes (18, 23, 36, 37).

First of all, we analyzed the data regarding serum levels of aspartate aminotransferase (AST) and alanine aminotransferase (ALT) to evaluate hepatic function in all three cases since these enzymes are considered indicators of liver cell injury (38). ALT is known to be a specific liver enzyme, even though it is expressed in other sites such as brain and skeletal muscle, as the levels of this enzyme in these tissues are low (39). In contrast, increased levels of AST can be found following damages in the cardiac and skeletal muscle tissues, which can explain why levels of these enzymes are found to be higher than ALT in most dengue cases (38, 39). The increase in transaminases is more accentuated in severe dengue cases of children when compared to adults (40). More than 10-fold rise in hepatic enzymes with greater levels of AST than ALT were observed before in an epidemiologic study from Brazil (38). This goes in line with our results, since all three cases presented levels of AST > 1000 U/L and ALT > 200 U/L, which may indicate a severe liver dysfunction and the possible development of acute liver failure (41). Several renal injuries were observed in kidney tissues of these three fatal cases of children in a previous study of our group (25). Therefore, multiorgan failure associated with profound shock observed in these patients could explain their poor outcome (42).

Nonetheless, it is worth mentioning that case one had a positive blood culture test for the bacteria *Pseudomonas aeruginosa* probably acquired in the hospital quarters. It is known that concurrent bacterial infection during hospitalization in cases of severe dengue can happen due to the patient’s debilitated condition, leading to systemic dysfunction by sepsis and worsening the clinical condition (43–45). The coinfection by DENV and this opportunistic pathogen has already been described in the literature (46, 47). The presence of the lipopolysaccharide (LPS), an important component located in the membrane of Gram-negative bacteria, including *Pseudomonas aeruginosa*, may play an important role in coinfections as LPS is capable of inducing the production of several pro-inflammatory cytokines through activation of the Toll-like receptor 4 (TLR4) (48). A previous study found that higher LPS levels were found associated with increased disease severity and pro-inflammatory cytokine profile in DENV-infected patients (49). Additionally, Chen and colleagues (50) demonstrated *in vitro* that DENV-infected monocytes and macrophages submitted to treatment with LPS, prolonged the viral replication and enhanced the production of INF-α. Therefore, together, these findings support the idea that in case one, the concurrent bacterial infection could have enhanced the infection and maintained the pro-inflammatory state, thus, contributing to the evolution of disease severity.

Previous researches of our group regarding dengue fatal cases of adults, found several lesions in liver tissues, including hemorrhage, edema and vascular congestion as well as areas of necrosis of hepatocytes and presence of lipid vesicles in hepatocytes cytoplasm (23). These findings are extremely important and similar to the results in the present study. Histopathological findings observed comprised severe circulatory dysfunction, characterized by vascular congestion in sinusoidal capillaries, edema disrupting the hepatic architecture and hemorrhage in all three fatal cases, indicating impairment of hepatic tissues and vascular permeability caused by DENV infection. These results were expected and are characteristic indicators of severe disease since the progression to worse clinical conditions in DENV infections are associated with increase in the vascular permeability and disruption of the endothelial barrier (51, 52). In addition, the development of shock state experienced by these patients could have been the result of either direct endothelial damage or systemic inflammation, which are common events during dengue infection (51, 53–55). Hepatocytes alterations such as vacuolar degeneration, a form of cell damage (56), and foci of necrosis were observed in these cases by histopathology analysis, which indicates organ injury and possible failure. Necrosis of hepatocytes and Kupffer cells were already demonstrated in other studies (15, 19).

We also observed other metabolic alterations in all DENV cases, which included abundant presence of multiple small (microsteatosis) or large (macrosteatosis) lipids droplets inside hepatocytes cytoplasm. Several studies have already correlated the development of steatosis with dengue infection, both in animal models and in human report cases (57–61). A study regarding alterations in fatty acid metabolism in DENV infections showed the presence of the viral capsid protein surrounding lipid droplets in DENV infected cells, suggesting a link between lipid metabolism and the encapsidation process during viral replication (62). It was already shown that DENV is capable of inducing autophagy of lipids droplets to release free fatty acids, thus, increasing β-oxidation, the main ATP-generating pathway, and consequently promoting viral replication (63–67). Therefore, it is possible that the diffuse lipid vesicles observed in all cases could have contributed to the spread of the virus in the hepatic tissue and subsequently other organs.

The liver tissue sections were also examined using Periodic Acid Schiff (PAS) stain, a special stain used to visualize glycogen deposits, especially in hepatic samples (68). The presence of PAS positive stain was observed in the parenchyma of control and DENV-infected tissues; however, absence or diminished PAS positive stain was observed in some areas of steatosis and necrosis in DENV-infected patients, revealing an apparently depletion of glycogen store in these sites. Reduction of glycogen stocks in hepatocytes infected with DENV serotype 4 was demonstrated before in a murine model (69).

The analysis of collagen deposition in areas of the hepatic tissues was also realized by staining sections with Picro Sirius Red, a special stain utilized to distinguish the different types of collagen fibers (I and III) and quantify liver fibrosis (70). An intense deposition of collagen of type I and III (red-yellow) was observed in perivascular areas of liver tissues of dengue cases, a process that may be associated to the degradation of specific components of the extracellular matrix, which can occur due to the action of proteolytic enzymes called metalloproteinases (71). Dysregulation of the extracellular matrix has already been seen in DENV infections (72). It is possible that these events may be associated with the expression of MMP-9, a key proteinase involved in extracellular matrix degradation (73), as this protein was detected in high levels in Kupffer cells and hepatocytes in all three cases. Elevated expression of this proteinase and rearrangements in extracellular matrix were noticed in previous works with DENV-infected kidney and pancreas tissues from children and adults (25, 30). Furthermore, a recent study suggests that NS1 protein could induce MMP-9 expression and interact with it, simultaneously acting on endothelial cells, promoting increased vascular permeability and consequently plasma leakage (74). The association of MMP-9 overexpression and progression of the disease severity have been investigated in the past years (75–79). In such a manner, it is conceivable that in these cases, MMP-9 may have also contributed to significant plasma leakage and worsened the clinical course of the patients, as it appears to be related with disease severity (80).

We tried to quantify the virus (DENV envelop gene sequence) using the primer set (forward 5′-TGGTTCCTAGACCTGCCGTTA-3′, Reverse 5′-TCTCTTTCTGTATCCAATT TGAT CCTT-3′ and 5′-FAM-CATGGCTACCCGGAGCGGACAC–TAMRA-3′) from the paraffin-embedded tissues; however, we were not able to detect the virus in any of the cases. This may be sometime due to the poor viral RNA quality in the embedded tissue section or any factors that affect the PCR reaction steps. Since the tissue samples were exhausted at the last and we were not able to repeat this experiment. However, we corroborated the severity of these three dengue cases based on the WHO classification (4). Then, by performing immunohistochemistry procedure, our group detected the NS3 protein in DENV-infected livers, a key viral antigen involved in the virus replication cycle (81, 82). The presence of the non-structural protein 3 was remarkably noticed in the cytoplasm of Kupffer cells, circulant macrophages and hepatocytes in hepatic parenchyma of the analyzed cases. These observations support the idea that, after infection, the virus was able to replicate in these sites, as this antigen appears when DENV starts its replication (83). Similar results were found in a mice model infected with dengue serotype 2 (DENV-2) (58). Moreover, dengue viral antigen was detected in Kupffer cells, macrophages and hepatocytes of DENV-infected adult patients (23, 29, 59, 84, 85), thus, corroborating with our results.

We also observed increased numbers of CD68+ cells infiltrated in hepatic parenchyma and inside sinusoidal capillaries of dengue cases, evidencing the presence of macrophages with characteristic hypertrophic morphology in these areas. The appearance of hyperplasic/hypertrophic macrophages in our results are extremely important as they are considered major targets of DENV infection as well as the primary source of inflammatory cytokines and consequently, they may play major roles in dengue pathogenesis (50, 86, 87). In addition, these cells are thought to be center pivots in the event of antibody-dependent enhancement, leading to the increased viral loads and therefore aggravating the clinical condition of patients, which could have occurred in the cases analyzed in this study (88–90). Furthermore, CD4^+^T and CD8^+^T cells were also detected in the portal vein, sinusoidal capillaries and composing the mononuclear infiltrate, where both lymphocytes could be responsible for producing cytokines and inflammatory mediators, as well as lysing target infected cells in response to DENV infection (91, 92). As our work demonstrated few lymphocytes CD4+, not making its quantification possible, we hypothesized that there is a more cytotoxic profile, with an intense activation of CD8+T cells in the cases analyzed, which has already been described elsewhere (93–95). This indicates that the cytotoxic response may play a key role in controlling DENV infections and could also be associated to the appearance of tissue damages and severe signs of the disease (96, 97). In line with our findings, previous reports of our group regarding dengue fatal cases of adults also revealed an increase in CD8^+^T and CD4^+^T cell subpopulations in liver tissues (24). Although CD4+ and CD8+ T lymphocytes have been shown to play major roles in controlling the viral infection through release of cytokines such as TNF-α or direct cytotoxicity, the complete function of these cells during DENV infections remains to be further investigated (98, 99).

The progression of mild illness to the severe forms of dengue are in close relationship with the enhancement of vascular permeability due to inflammatory cytokines and mediators produced by cells in response to viral infection (100, 101). Thus, one of the goals in this research was to scrutinize the cytokine profile in the three dengue cases, which was characterized by the expression of remarkable markers associated with vascular dysfunction, including TNF-α, one of the main pro-inflammatory cytokines involved in dengue immunopathogenesis (102). Overexpression of TNF-α was mainly observed in the endothelium of sinusoidal capillaries and by circulant macrophages infiltrated in liver-infected tissues, which goes in line with previous results of our group (24). The presence of these infiltrating macrophages expressing TNF-α has already been shown to stimulate production of reactive oxygen species (ROS), induce endothelial damage and consequently contribute to hemorrhage development, a significant outcome observed in our results (103, 104). Indeed, the loss of balance between oxidants and antioxidants agents may lead to greater complications during dengue infection (105). In line with that, it is the study conducted by Soundravally and colleagues, who found increased levels of TNF-α and malondialdehyde (MDA), a biomarker of oxidative stress, in the plasma of patients with dengue hemorrhagic fever (DHF) and dengue shock syndrome (DSS) (106). The researchers also revealed a positive association between these two molecules, thus, evidencing that the oxidative stress induced by DENV, may contribute to the release of inflammatory mediators (106). Furthermore, endothelial cells subjected to TNF-α exposure *in vitro* also revealed increased vascular permeability (107), on the other hand, inhibition of this cytokine has been linked to decreased disease severity in animal models (108), highlighting the importance of this inflammatory mediator in dengue infections.

Furthermore, it was possible to notice increased numbers of RANTES-producing cells in the mononuclear infiltrates around the centrilobular vein and perivascular areas as well as circulant macrophages, which may have led to the increased infiltrates of lymphocytes and macrophages observed at the inflamed sites, since this chemokine acts as an important mediator to the leukocyte recruitment (109, 110). Following this reasoning, a previous study regarding hepatic tissues found high expression of this chemokine in dengue fatal cases (24), however, low serum levels of RANTES were reported in the blood of DENV-infected patients (111). It is also possible that RANTES could have a role in modulating the expression of the vascular endothelial growth factor (VEGF), another mediator involved in alterations of the vascular permeability (112). Thus, in the present work we investigated the expression of VEGFR-2 (vascular endothelial growth factor receptor 2), the receptor of VEGF, which was found increased in all three dengue cases, being mainly expressed by hepatocytes, endothelial cells and macrophages. Endothelial cells infected with DENV *in vitro* showed that DENV could up-regulate the expression of VEGFR-2 in the surface of these cells, and thus, promote the stimulation by VEGF (113). In addition, VEGFR-2 and both its factor have already been associated with severe plasma leakage in DENV infection (114, 115). Finally, we also detected the expression of VCAM-1 (vascular cell adhesion molecule 1) in the endothelium of sinusoidal capillaries in hepatic tissues, which could have occurred due to stimulation by VEGF, since this mediator is able to induce expression of several adhesion molecules in the endothelium (116). Nonetheless, VCAM-1 interacts with integrins on the surface of many leukocytes, assisting cell migration and leukocyte recruitment during the inflammatory process, thus, favoring the occurrence of cell infiltration (117).

## 5 Conclusion

Our findings evidence the presence and replication of DENV in liver tissues, which leads to a strong pro-inflammatory response, consequently resulting in severe histopathological alterations and functional damage. Qualitative studies showing the immunopathologic basis of DENV infection in children is very rare, and therefore, the findings in the present work provided valuable insights about the complex interplay between cells and cytokines expressed in DENV infection, especially in this specific population. The research also adds relevant information for the “cytokine storm” theory and how it can be associated with the fatal outcome experienced by these patients.

## Data availability statement

The original contributions presented in the study are included in the article/**Supplementary Material**. Further inquiries can be directed to the corresponding author.

## Ethics statement

The studies involving human participants were reviewed and approved by Ethics Committee of the Oswaldo Cruz Foundation/FIOCRUZ (CAEE: 47525115.3.0000.5248). Written informed consent to participate in this study was provided by the participants’ legal guardian/next of kin.

## Author contributions

Conceptualization: KR and MP. Methodology: all authors. Investigation: FA, CA, LM, KR and MP. Writing—original draft Q20 preparation: FA, LM and KR. Writing—review and editing: NS, KR and MP. Supervision: KR and MP. Project administration: MP. Funding acquisition: MP. All authors have read and agreed to the published version of the manuscript.
