# Supplementary material for: Liver immunopathogenesis in fatal cases of dengue in children: detection of viral antigen, cytokine profile and inflammatory mediators
Source: Front Immunol. 2023 Jun 30;14:1215730. doi: 10.3389/fimmu.2023.1215730 (PMC10348746; doi:10.3389/fimmu.2023.1215730)
Supplement: Supplementary file 1 [file DataSheet_1.docx]

**Case 1 - Hemorrhagic dengue**

Boy, melanodermic, 7 years and 8 months old, born on 5/10/2000 in Rio de Janeiro, hospitalized at HMJ on 1/30/2008. On 1/29/2008, after five days of high fever, nausea and vomiting, the mother sought medical care at a health center, being medicated with amoxicillin for diagnosis of “infection.” Local blood count showed: hematocrit: 39.3%; leukocytes: 5,280/μl; red cells: 4.57 x 10 3 /μl; platelets: 70.8 x 10 3 /μl. Not improving, on 1/30/2008, the mother went to the medical assistance unit, where another blood count showed: hematocrit: 45.9%; leukocytes: 8,390/μl; red cells: 5.41 x 10 3 /μl; platelets: 17.9 x 10 3 /μl; the presumptive diagnosis of dengue was made, and hospital admission was arranged.

**Notes:** The boy had chickenpox at age 4 and was born by normal delivery, at term, fraternal twins. He lives in a brick house, with good sanitary conditions, with running water, however, the family lives with chickens and dogs and next to the residence, there is an open sewer.

**Physical examination:** Marked protein-calorie malnutrition, weighing 15 kg; pale; hypohydrated, acyanotic, anicteric, with slow capillary filling, without petechiae or ecchymosis on the skin. With discreet gingival bleeding. Cardiovascular System: normal cardiac auscultation; low-amplitude arterial pulses; blood pressure of 80x50 mmHg. Respiratory system: pulmonary auscultation with universally audible vesicular murmur, rude, without adventitious sounds; 29 breaths per minute. Abdomen: painful on deep palpation, with sign of peritoneal irritation. Liver 2 cm from the right costal margin. He had moderate hematemesis after abdominal palpation. Child in serious condition, hemodynamically unstable. Admitted to the ICU: received intravenous hydration; platelet concentrate and cardiovascular and respiratory monitoring. Chest radiography showed large pleural effusion right.

**Evolution:**

**1/31/2008:** serious condition; hydrated; pale; afebrile (axillary temperature 36.5°C); tachypneic (36 irpm); 105 heartbeats per minute; blood pressure of 105x61 mmHg; with bilateral periorbital edema; little diuresis (240 ml). Pulmonary auscultation with slightly reduced breath sounds at the right base. He vomited in “coffee grounds” three times.

**2/1/2008:** Severe general condition, awake, prostrate, pale, acyanotic, icteric, fever of 39.4oC, complaining of abdominal pain and dyspnea. Tachypneic (41 bpm) and tachycardic (130 bpm). Blood pressure of 90x46 mmHg. Pulmonary auscultation with reduced breath sounds in right hemothorax due to pleural effusion, leading to respiratory effort. A small pleural effusion was observed on the left side. He underwent right thoracentesis, with the output of 247 ml of citrine yellow pleural fluid, favoring improvement in breathing. Large amount of greenish liquid diarrhea in the morning. Abdomen globose, ascitic, with mild hepatomegaly. Oliguric (diuresis of 96 ml/kg/h). On ventilatory support, with bleeding from the nostrils after aspiration. Anemia and thrombocytopenia.

**2/2/2008:** In ventilatory prosthesis, sedated, hydrated, afebrile. Hepatosplenomegaly. At night, he did not react to stimuli. Dry mucous membranes, pale, thin pulse. Blood pressure of 81x43 mmHg. Tachycardic (122 bpm). ascites. Anemia and thrombocytopenia. Positive blood culture for *Pseudomonas aeruginosa.*

**2/3/2008:** Very serious general condition. Afebrile Hemodynamically unstable. Flushed, with periorbital edema in hands and feet. tachycardic. Heart rate ranging from 103 to 149 bpm. Blood pressure ranging from 74x34 mmHg to 112x74 mmHg. Variable respiratory rate from 20 to 39 bpm. Globular abdomen, ascitic. Liver 5 cm from the right costal margin. Spleen not palpable. Right pleural effusion. Oliguric (2.5 ml/kg/h). With anemia and thrombocytopenia. Epistaxis. Metabolic acidosis.

**2/4/2008:** Severe, afebrile, in anasarca, pale, acyanotic, anicteric. ascites. Mean arterial pressure [(2PD + PS)/3], ranging from 63 to 94 mmHg. Tachycardic (132 to 179 bpm). Oliguric (1.6 ml/kg/h). With bleeding coming out of the orotracheal tube. He presented bradycardia (38 bpm), being submitted to cardiac massage and intravenous adrenaline.

**2/5/2008:** Extremely serious, vasodilated, shocked. ascites. Anasarca. Thin pulses, cold extremities, slowed capillary perfusion. Tachycardic (122 to 146 bpm). Mean arterial pressure ranging from 73 to 90 mmHg. Axillary temperature ranging from 35oC to 37.5ºC. Respiratory Distress Syndrome.

**2/6/2008:** Serious. sedated. hypohydrated. acyanotic. jaundice ascitic. Anasarca. Fine pulses. Axillary temperature: 36 – 37.7ºC. Heart rate from 106 to 147 bpm. Mean arterial pressure: 51 to 93 mmHg. Respiratory rate from 25 to 40irpm. Respiratory Distress Syndrome (chest radiography with diffuse bilateral pulmonary infiltrate and pleural effusions). Oliguria (1.4 to 0.33 ml/kg/h). Increased nitrogenous slag. Acute renal failure. Hemodialysis requested. Refractory shock; septic.

**2/7/2008:** Same as the previous clinical status, with axillary temperature from 35 to 37.5ºC; heart rate from 111 to 145 bpm; mean arterial pressure from 73 to 105 mmHg; respiratory rate of 28 to 30 bpm. On hemodialysis. Refractory shock.

**2/8/2008:** Same as previous clinical conditions, refractory, septic shock, with axillary temperature of 35 to 37ºC; heart rate from 112 to 159 bpm; mean arterial pressure from 91 to 108 mmHg; respiratory rate of 20 to 32 bpm. On hemodialysis. Echocardiogram: systolic and diastolic myocardial dysfunction, with volume overload. Skin lesions suggestive of vasculitis in the left foot.

**2/9/2008:** Same as previous clinical states. Axillary temperature of 35ºC; heart rate from 115 to 124 bpm; blood pressure from 111 x 71 mmHg to 125 x 81 mmHg; respiratory rate of 25 to 39 bpm. Skin lesions suggestive of vasculitis on hands and feet. Marked hepatomegaly. On hemodialysis.

**2/10/2008:** Same as above. Sepsis. SARA. Refractory shock. hemodialysis. Peripheral vasodilation. Vasculitis in digital extremities in hands and feet. Axillary temperature from 36 to 37.6ºC; heart rate from 115 to 130 bpm; mean arterial pressure from 59 to 85 mmHg; respiratory rate from 18 to 30irpm. Chest radiograph with bilateral gross infiltrate. Pulmonary auscultation with coarse vesicular murmur, sub-crepitate rales in bases and diffuse rumbling. Cardiac auscultation with systolic murmur at the left sternal border. Diuresis: 2.5 ml/h. Blood clot exit through the orotracheal tube. Eliminated two ascaris.

**2/11/2008:** Same as above. Sepsis. SARA. Refractory shock. hemodialysis. Urinary output: 0.04ml/kg/h. 15ml/day. Axillary temperature: 35ºC to 37ºC. Heart rate: 110 to 120 bpm. Mean blood pressure: 78-110 mmHg. Myocardial dysfunction and volume overload. Respiratory rate: 23 – 52 bpm. Tachypnea. Pleural effusions. Abdominal USG: Moderate ascites. Bleeding from the nostrils.

**2/12/2008:** Same as above. Sepsis. SARA. Refractory Shock. Blood dyscrasia. Axillary temperature: 35.4 to 38ºC. Heart rate: 107 – 132 bpm. Blood pressure: 151x78 mmHg; 95x53mmHg. Respiratory Rate: 26-72irpm. hemodialysis. Anuria. Orotracheal tube with blood clot outlet.

Blood pressure drop. bradycardia. No arterial pulses. Non-responsive to drugs (vasoactive amines). Death recorded at 3:15 pm.

**2/13/2008:** Submitted to autopsy.

**Transfusions:**

**1/30/2008:** Concentrate of platelets and red blood cells.

**2/1/2008:** Platelet and red blood cell concentrate; plasma (12/12h) 2/2/2008: Concentrated red blood cells

**4/2/2008:** Plasma and Platelet Concentrate. 2/5/2008: Plasma (12/12h)

**2/6/2008:** Platelet Concentrate; and plasma (12/12h) 2/7/2008: Plasma 12/12h

**8, 9 and 10/2/2008:** Concentrate of red blood cells; and plasma (12/12h).

**Laboratory tests: Biochemistry and Hemogram (2008)**

|  | 1/30 | 2/1 | 2/2 | 2/3 | 2/5 | 2/6 | 2/8 | 2/10 | 2/11 | 2/12 | Limits |
| --- | --- | --- | --- | --- | --- | --- | --- | --- | --- | --- | --- |
| Urea | 34.6mg/dl | 27mg/dl | 22.2mg/dl | 34.6mg/dl | 80.0mg/dl | 111mg/dl | 17.3mg/dl | 63.7mg/dl | 40.7mg/dl | 95.5mg/dl | 10-50 mg/dl |
| Creatinine | 0.947mg/dl | 0.639  mg/dl | 0.110  mg/dl | 0.947  mg/dl | 1.24-1.74  mg/dl | 0.331  mg/dl | 0.992  mg/dl | 23.8  mg/dl | 0.531  mg/dl | 1.24  mg/dl | 0.300-0.500mg/dl |
| Glucocose | 87.3mg/dl | 111mg/dl | 80mg/dl | 110mg/dl | 102mg/dl | - | - | - | - | - | 70-110mg/dl |
| Albumin | 3.06g/dl | 2.64g/dl | 3.16g/dl | 3.06g/dl | - | - | - | - | 3.77g/dl | 3.66g/dl | 3.50-4.80g/dl |
| Total protein | 5.26g/dl | 4.56g/dl | 4.72g/dl  5.15g/dl | 5.26g/dl | 6.62g/dl | - | - | - | - | 6.81g/dl | 6.10-7.90g/dl |
| AST/TGO | 640U/l | 2117U/l | 1720U/l | 640U/l | 291U/l | 830U/l | - | 189U/l | - | 146U/l | 0 -32 U/I |
| ALT/TGP | 175U/l | 385U/l | 328U/l | 175U/l | 291U/l | 137U/l | - | 68.5U/l | - | 59.7/l | 0-31U/I |
| GT range | 75.9U/l | 85U/l | - | 75.9U/l | 66U/l | 73.4U/l | - | 76.5U/l | - | 63.7U/l | 11-50U/I |
| Phosphatase  alkaline | - | - | - | - | 671U/I | 1306U/I | - | 838U/I | - | 476U/I | 65-645U/I |
| Total bilirrubin | 2.42mg/dl | - | - | 2.42mg/dl | 7.39mg/dl | 14.6mg/dl | - | 22.8mg/dl | - | 18.6mg/dl | 0-1mg/dl |
| Direct bilirrubin | 1.29mg/dl | - | - | 1.29mg/dl | 4.85mg/dl | 9.88mg/dl | - | 14.8mg/dl | - | 13.5mg/dl | 0-0.20mg/dl |
| Calcium | 7.28mg/dl | 7.10mg/dl | 7.89mg/dl | 7.28mg/dl | 8.29mg/dl | - | 9.95mg/dl | 9.46mg/dl | 10.7mg/dl | 9.09mg/dl | 8.5-10.5mg/dl |
| Magnesium | 1.06mg/dl | - | 1.01mg/dl | 1.06mg/dl | 1.96mg/dl | 1.53mg/dl | - | 1.50mg/dl | 1.76mg/dl | - | 1.90-2.50mg/dl |
| Phosphate | 3.64mg/dl | - | 1.50-2.52mg/dl | 3.64mg/dl | - | 5.60mg/dl | - | 0.692  mg/dl | - | 2.09mg/dl | 4-7mg/dl |
| Sodium | - | 125meq/I | 134meq/l | 133meq/l | 135meq/l | 136meq/I | 139meq/l | 137meq/l | 134meq/l | 138meq/l | 136-146meq/I |
| Potassium | - | 4.6meq/I | 4.1meq/l | 4.4meq/l | 3.6meq/l | 1.1meq/I | 3.7meq/l | 4.0meq/l | 3.6meq/l | 4.7meq/l | 3.5-5.0meq/I |


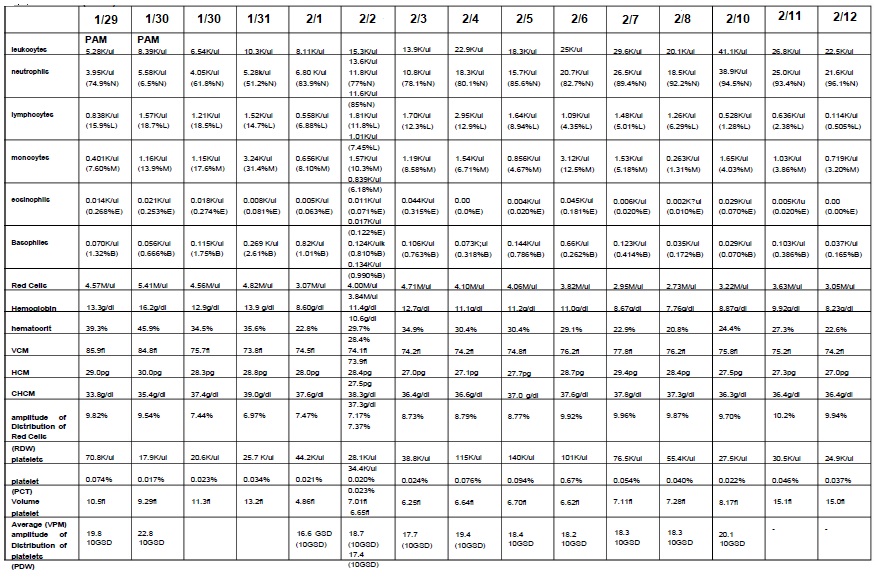


| **Hemogram limits:** |  |
| --- | --- |
| Leukocytes | 4.60 to 10.2 K/ul |
| Neutrophils | 2.00 to 6.90 K/ul (37.0 to 80.0%N) |
| Lymphocytes | 0.600 to 3.40 K/ul (10.0 to 50.0%L) |
| Monocytes | 0.00 to 0.900 K/ul (0.00 to 12.0%M) |
| Eosinophils | 0.00 to 0.700 K/ul (0.00 to 7.00%E) |
| Basophils | 0.00 to 2.00 K/ul (0.00 to 2.50%B) |
| Red cells | 4.04 to 6.13M/ul |
| Hemoglobin | 12.0 to 18.1 g/dl |
| Hematocrit | 36.0 to 53.7% |
| VCM | 80.0 to 97.0fl |
| HCM | 27.0 to 31,2pg |
| CHCM | 31.8 to 35.4g/dl |
| RDW | 11.6 to 14.8% |
| Platelets | 142,000 to 424,000/ul |

**1/29/2008** (PAM) 12h57min: Blood count: leukopenia, lymphopenia, hyperchromic anemia and thrombocytopenia.

**1/30/2008** (PAM)11h33min: Blood count: neutrophilia, lymphopenia, thrombocytopenia.

**1/30/2008** HMJ 21:12: CBC: monocytosis, hyperchromic microcytic anemia, thrombocytopenia.

**1/31/2008** HMJ 14:35: CBC: leukocytosis, monocytosis, basophilia, hyperchromic microcytic anemia, thrombocytopenia.

**2/1/2008** HMJ 11:51 am and 11:01 pm: CBC: monocytosis, lymphopenia, hyperchromic microcytic anemia, thrombocytopenia.

**2/2/2008** HMJ 9:50 am and 6:07 pm: CBC: leukocytosis, neutrophilia, monocytosis, hyperchromic microcytic anemia, thrombocytopenia.

**2/3/2008** HMJ 2:51 pm: CBC: leukocytosis, neutrophilia, monocytosis, hyperchromic microcytic anemia, thrombocytopenia.

**2/4/2008** HMJ 11:32 am: Blood count: leukocytosis, neutrophilia, monocytosis, hyperchromic microcytic anemia, thrombocytopenia.

**2/5/2008** HMJ 2:43 pm: Blood count: leukocytosis, neutrophilia, monocytosis, hyperchromic microcytic anemia, thrombocytopenia.

**2/6/2008** HMJ 10:46 am: Blood count: leukocytosis, neutrophilia, monocytosis, hyperchromic microcytic anemia, thrombocytopenia. ESR: 31mm/h

**2/7/2008** HMJ 10:52pm: Blood count: leukocytosis, neutrophilia, monocytosis, hyperchromic microcytic anemia, thrombocytopenia.

**8/2/2008** HMJ 16:17: Blood count: leukocytosis, neutrophilia, hyperchromic microcytic anemia, thrombocytopenia.

**2/10/2008** HMJ 5:49 pm: Blood count: leukocytosis, neutrophilia, lymphopenia, monocytosis, hyperchromic microcytic anemia, thrombocytopenia.

**2/11/2008** HMJ 1:37 pm: Blood count: leukocytosis, neutrophilia, monocytosis, hyperchromic microcytic anemia, thrombocytopenia.

**2/12/2008** HMJ 12:42 pm: Blood count: leukocytosis, neutrophilia, lymphopenia, hyperchromic microcytic anemia, thrombocytopenia.


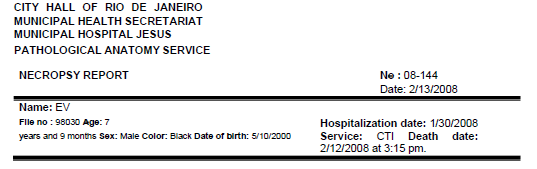


**Cause of death**: extensive bilateral pulmonary hemorrhage

**Main diagnosis**: Hemorrhagic dengue (Confirmed by positive serology result for dengue IgM antibodies using Dengue IgM kit - Elisa Capture – PanBio)

**Other diagnoses:**

1. Lungs: Bilateral pneumonitis, with foci of bronchiolitis. Congestion and edema. Extensive bleeding with foci of necrosis. Pleural effusions.

2. Focal chronic tracheitis and laryngitis with areas of mucosal squamous metaplasia. Hemorrhagic content in the lights.

3. Mild focal pericarditis. Pericardial effusion. Myocardial congestion.

4. Tongue with foci of hemorrhage. Secondary hypoplasia of lymphoid tissue.

5. Mild chronic esophagitis and congestion.

6. Superficial mild chronic gastritis in body and pyloric antrum. Congestion.

7. Chronic duodenitis with some lymphoid follicles.

8. Jejunum and ileum with secondary hypoplasia of mucosa-associated and submucosal lymphoid tissue. Ascariasis.

9. Nonspecific chronic colitis and proctitis.

10. Moderate ascites.

11. Nonspecific, focal, chronic sialoadenitis in submandibular salivary glands.

12. Subacute pancreatitis with foci of intralobular edema and acinar necrosis.

13. Hepatomegaly. Acute hepatitis with steatosis, foci of necrosis and centrilobular hemorrhage. Intrahepatic cholestasis. Hemorrhagic shock.

14. Spleen, thymus, lymph nodes and vermiform appendix: Secondary hypoplasia of lymphoid tissue. Congestion. thymic atrophy. splenomegaly.

15. Thyroid: Bleeding into soft tissues adjacent to the thyroid.

16. Pituitary and adrenals: Congestion.

17. Kidneys: Proximal tubular alterations resulting from hydroelectrolytic disturbance. Congestion.

18. Bladder: Congestion of the mucosa.

19. Testicles and epididymis: Congestion. Foci of hemorrhage in the testicular interstitium. Seminiferous tubules, in foci, with psammomas (calcification).

20. CNS: Hypoxic-ischemic lesions mainly in the brainstem and cerebellum. Slight dilation of the lateral cerebral ventricles.

21. Bone marrow: Relative cellular hyperplasia, with a predominance of immature cells, associated with loose aggregates of lymphocytes (many with apoptosis). Megakaryocytic hypoplasia and dysplasia.

22. Pleural fluid (08-161): Autolyzed material. Sparse lymphocytes and squamous epithelial cells. Absence of neoplastic cells.

23. Ectoscopy: Weight loss (protein-caloric malnutrition). Jaundice. Bilateral cervical edema predominantly juxtamastoid. Venipuncture marks on the neck, subclavicular regions and upper and lower limbs. Soft tissue hematoma of the right cervical and subclavian regions. Left heel plantar region with hemorrhage. Dorsum of right wrist with erosion/ulceration and hemorrhage. Free from hypostasis on the back. rigor mortis.

Fernando Colonna Rosman

CRM 5252023-5

**Case 2 - Hemorrhagic dengue**

Girl, 9 years old, 11 months, 28 days old, born on 4/27/2001, born in Itaboraí, RJ, on 4/23/2011 started to have fever (38ºC, 39.5ºC) and general malaise, being taken to the Health Center on 4/24/2011, where a blood count, at 10:27 am, revealed a hematocrit of 36.8% (N=32.7 to 39.6%); Red cells 4.48 million/ mm3 (N= 4.0 to 4.9 million/mm3 ), Hemoglobin of 12.9g/dl (N= 10.9 to 13.3g/dl), MCV of 82.1fl (N = 77 to 85fl), HGM 28.8pg, CHGM 35.1%; Leukocytes totaling 2,700/mm3 (N= 5.0 to 14.5/mm3 ) (2% rods with 54/mm3 (N= 3/mm3 ), 75% segmented with 2,025/mm3 (N= 48 to 52 /mm3), 15% lymphocytes with 405/mm3 (N=37 to 39/mm3), 8% monocytes with 216/mm3 (N=4 to 5/mm3 ); Platelets totaling 76,000/mm3 (N=150,000 to 550,000/mm3); and Blood count, on 4/25/2011, at 5:50 am, showed Hematocrit of 33.4% (N= 37 to 47%) Leukocytes totaling 2,200/mm3 (N= 4,500 to 10,000/mm3) and Platelets totaling 22,000 /mm3 (N=150 mil to 450 mil/mm3). On physical examination, she presented with drowsiness and prostration, but cooperating with the examination, dehydration, pale, eupneic (respiratory rate of 22irpm, 20irpm, 24irpm), acyanotic, tachycardic (heart rate of 130bpm), blood pressure of 90x60mmHg and 90x50mmHg; axillary temperatures of: 36.2ºC, 38ºC, 37ºC and 36.8ºC. Normal cardiac and chest auscultation. No sign of meningeal irritation. Globular abdomen, with painful hepatomegaly, peristalsis present. Spleen not palpable. Petechiae was observed on the face and lower limbs. He was treated with ranitidine, dipyrone, replacement and maintenance hydration with saline and glucose solutions and with the addition of sodium chloride and potassium chloride. She was transferred to the hospital on 4/25/2011.

**Hospital evolution (ICU):**

**4/25/2011:** Spent 10 hours and 40 minutes in the ICU, receiving hydroelectrolytic replacement and hemodynamic control, with usual medication (albumin, adrenaline, noradrenaline, furosemide, sodium bicarbonate). At 2 pm, she was awake, lucid, oriented, pale, with slight swelling of the eyelids, and with tachypnea (O2 saturation of 96%, with nasal catheter), blood pressure of 79x63 mmHg, 79x50mmHg, heart rate of 128 bpm. At 16:00 she was awake, saying she was fine, with a heart rate of 124 bpm, blood pressure of 88x70x(55)mmHg, respiratory rate of 28-30irpm, diuresis (12:00 to 16:00h) of 565ml. At 18:00 she was awake, with blood pressure of 104x86x63mmHg, heart rate of 160bpm, respiratory rate of 28-32irpm, O2 saturation of 96%. At 18:35, he had a tachycardia of 180 bpm and a mean blood pressure of 36 mmHg. At 7 pm, mean arterial pressure was 80mmHg, heart rate was 160bpm, and O2 saturation was 96%. At 7:35 pm, heart rate was 160 bpm and blood pressure was unmeasurable (cold shock). From 7:45 pm , he presented bradycardia and a drop in blood pressure and a non-palpable peripheral pulse, and external cardiac massage was performed. She was medicated with adrenaline, leading to a tachycardia of 140bpm and a mean blood pressure of 35-40mmHg. Despite efforts to maintain cardiovascular and respiratory functions, through mechanical ventilation, maintenance of blood volume, acid-base balance, with the administration of vasoactive amines, albumin, sodium bicarbonate, etc., he had a mean arterial pressure of 35 mmHg and a fall heart rate from 160bpm to 85bpm, 60bpm, to irreversible cardiac arrest, with death being recorded at 10:25 pm.

**4/26/2011:** Complete necropsy.


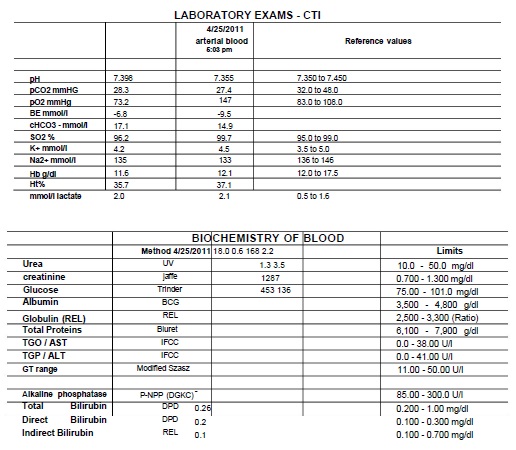


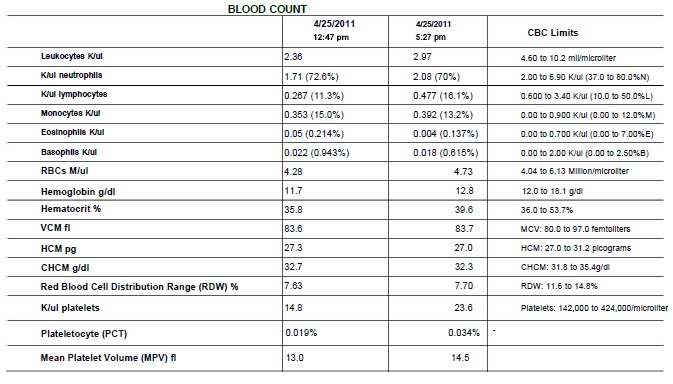


**03/25/2011:** Leukopenia, neutropenia, lymphopenia, anemia, thrombocytopenia.


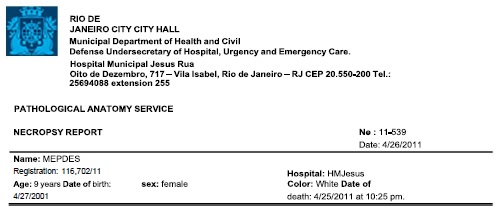


**Cause of Death:** Hemorrhagic shock. Multiple organ failure syndrome.

**Main diagnosis:** Dengue (Confirmed by positive result using dengue NS1 Kit: immunochromatographic test for the detection of the NS1 antigen of the dengue virus).

**Other diagnoses:**

1. Lungs: Bronchitis, pneumonitis (foci), congestion, edema, hemorrhage (multifocal), anthracosis, erythrophalkemia.

2. Pleura: Marked bilateral effusion.

3. Larynx: Laryngitis with lymphoid follicles, mucosal edema and congestion.

4. Trachea: Mild tracheitis.

5. Heart: Vascular congestion. Small focus of septal subendocardial hemorrhage in the left ventricle.

6. Submandibular salivary glands: Chronic nonspecific sialoadenitis.

7. Tongue: Lymphoid follicles, from the posterior region, with hemorrhage.

8. Esophagus: Esophagitis with lymphoid follicles. Parietal hemorrhage, focal, in the lower third.

9. Stomach: Vascular congestion, foci of hemorrhage and lymphoid follicles in the mucosa.

10. Duodenum, jejunum, ileum and large intestine: Vascular congestion and lymphoid follicles in the mucosa.

11. Liver: Hepatomegaly. Marked passive hyperemia with hemorrhage and/or necrosis in zones 2 and 3 of the lobules, secondary to acute ischemia (acute circulatory system). Reactive hepatitis. Erythrophakemia.

12. Spleen: Splenomegaly. Erythrophakemia. Red pulp congestion, with foci of extramedullary hematopoiesis and multifocal hemorrhage.

13. Mediastinal and mesenteric lymph nodes: Nonspecific reactive histiocytic hyperplasia. Mild anthracosis in the mediastinum.

14. Vermiform appendix: Reactive follicular hyperplasia.

15. Thymus: Vascular congestion. Hassall's corpuscles with calcification.

16. Bone marrow: Relative cellular hypoplasia with few differentiated cells (predominance of immature cells), associated with megakaryocytic dysplasia. Erythrophakemia.

17. Kidneys: Glomerular and interstitial vascular congestion. Erythrophakemia. Tubular alterations resulting from hydroelectrolytic disturbance. Focal acute tubular necrosis.

18. Bladder: Chronic cystitis with foci of necrosis and hemorrhage in the bladder neck mucosa.

19. Ovaries: Cystic follicles.

20. Peritoneal cavity: Moderate ascites.

21. Anasarca. Pallor. Vascular puncture marks on the upper limbs. Rigor mortis and free of hypostasis on the back.

Fernando C. Rosman

CRM 52 52023-5

**Case 3 - Hemorrhagic dengue**

Boy, aged 10 years, 8 months and 20 days, born on 7/30/2001, born in Rio de Janeiro, RJ, presented, at night, fever of 38.5oC and frontal headache, on 4/14/2012 before seek medical attention at the Emergency Care Unit, as the day before, he had prostration, non-food vomiting, and a fever of 38oC. He received initial medical treatment at the UPA and was referred to the Dengue Center and then to hospital, after blood count results on 4/18/2012, with leukopenia, thrombocytopenia, suspected of Dengue.

**4/18/2012:** On physical examination, on admission at 1:30 pm, he was eutrophic (weight 28,500g), hydrated, anicteric, acyanotic, with axillary temperature of 37oC, respiratory rate of 40irpm, with reduced vesicular murmur in the lung bases, normal cardiac auscultation, blood pressure, lying, 110X62mmHg and sitting, 97x76mmHg, heart rate of 112bpm, filiform peripheral pulses; globular abdomen, painful on superficial and deep palpations. Glascow level of consciousness 15. Strofulous scarring of lower limbs. She received rapid, maintenance venous hydration, with fluid-electrolyte control, and blood was collected for laboratory tests. At 1:30 pm, he was prostrate, tachydyspneic, with an Oxygen Saturation of 94%; abdominal pain and distension, pallor, hydrated (no diuresis); lying blood pressure 104x52mmHg, heart rate 108bpm. Transferred to CTI. Physical examination at the ICU showed drowsiness, swelling, rash, hydrated, ruddy, tachypneic, normal cardiac auscultation, chest auscultation with reduced vesicular murmur in the lung bases, globular abdomen (ascites?) and painful. At 4:30 pm with blood pressure of 105x75mmHg, heart rate of 116bpm and Oxygen Saturation of 96%. Venous blood tests: pH 7.242 (N=7.350 to 7.450); PCO2 44.3mmHg (N=35.0 to 45.0); PO2 35.8mmHg (N=80.0 to 100.0); BE -8.5mmol/l, cHCO3 - 18.7mmol/l; SO2 56.8% (N=75.0 to 99.0); K+ 2.54mmol/l (N=3.50 to 4.5); Na+ 146.4mmol/l (N=135.0 to 148.0); Cl 105.3mmol/l (N=98.0 to 107.0); Hb 15.0g/dl (N=11.5 to 17.4); Ht 41.6% (N=35.0 to 50.0); O2Hb (oxyhemoglobin) 54.9% (N=95.0 to 99.0); HHb (deoxyhemoglobin) 41.7% (N=1.0 to 5.0); glucose 204mg/dl (N=60 to 110mg/dl); Lactate 5.8mmol/l (N=0.4 to 2.23)

**4/19/2012:** Acyanotic, with dyspnea. Orotracheal intubation was performed. Severe, shocked, pulseless, mechanically ventilated (33x8x100%x17), SatO2 96%, hypohydrated; anicteric; acyanotic; absent central and peripheral arterial pulses; isochoric, miotic and photoreactive pupils. Axillary temperature from 36 to 35.6 oC; Heart rate from 117 to 121 bpm; Mean Blood Pressure from 90 to 88mmHg; Respiratory rate from 32 to 62 bpm; Diuresis of 3.3ml/kg/h (oliguria). Auscultation of the chest with abolished breath sounds in the lower 2/3 of the lungs bilaterally, normal cardiac auscultation, heart rate of 106bpm, blood pressure of 76x47mmHg. Distended, painful, ascitic abdomen. Liver 5 cm from the right costal margin. Subjected to conventional treatments for hydroelectrolytic and acid-base control, noradrenaline, etc. At 11 am with anuria. Central pulses present and peripheral pulses absent. Blood pressure of 180 x 110mmHg. At 12 pm with diuresis of 0.73ml/kg/h and blood pressure of 140x110mmHg. severe acidosis. Plasma transfusion 10ml/kg. At 12:30 pm with active bleeding from the gastric tube and hematuria. At 16:00, wide pulses, anasarca, blood pressure 120x71mmHg. At 18:30, with heart rate up to 52 bpm, he went into cardiorespiratory arrest; performed external cardiac massage, ventilation with ambu and administered adrenaline, atropine and bicarbonate for 20 minutes, without effectiveness. A bilateral relief thoracic puncture was performed (970ml of pleural fluid was drained). Heart rate return to 156bpm. Blood pressure of 141x76mmHg. SatO2 90%. Median and photoreactive pupils. Right femoral vein puncture was performed and hemodialysis catheter was placed, which started at 6:50 pm. At 20:00, very serious, blood pressure of 65x55mmHg; non-palpable peripheral pulses; weak central pulses; pupils tending to miosis and poorly photoreactive. Good chest expansion. Universally audible vesicular murmur. Normal cardiac auscultation. Ascitic abdomen. At 20:20, blood pressure of 125x85mmHg. At 21:10, blood pressure and 45x40mmHg, heart rate of 100bpm. At 21:20, blood pressure of 65x45mmHg. At 21:50, blood pressure of 47x43mmHg. At 23:20, blood pressure and 90x45mmHg. Blood ultrafiltration (hemodialysis) was started at 11 pm.

**4/20/2012:** 3h, blood pressure 75x57mmHg, heart rate 150bpm. At 4:10 am, blood pressure 92x46mmHg, heart rate 140bpm. At 5:50 am, with cardiorespiratory arrest, resuscitation maneuvers and administration of adrenaline, bicarbonate and glucose were performed. At 7 am, his death was recorded and he went under a complete autopsy.


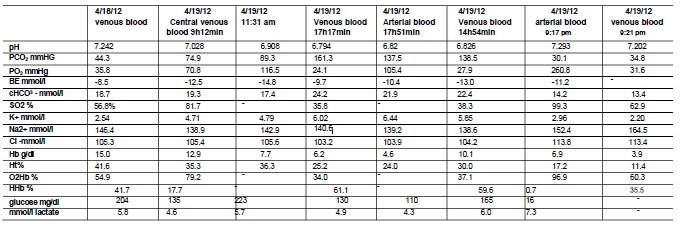


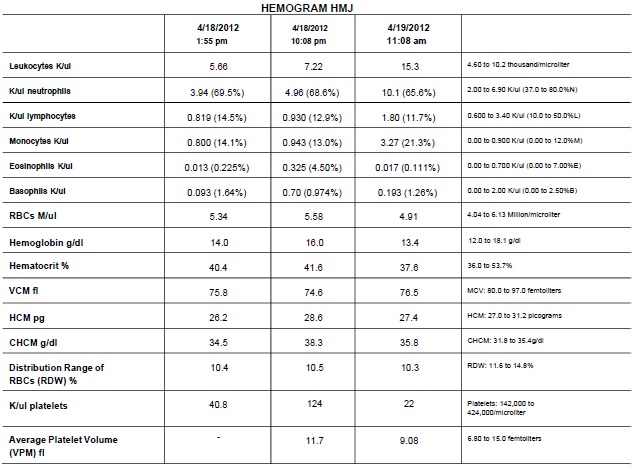


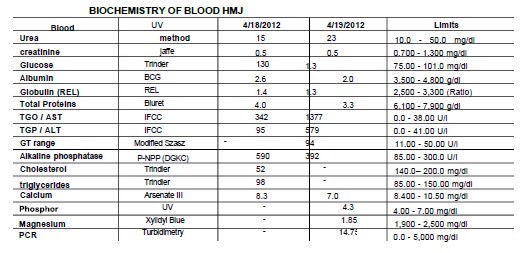


Patient transferred on 4/18/2012 from CMS Masao Goto to Hospital Municipal Jesus with hematocrit of 40% and 40,000 platelets per mm3 , prostrate, tachydyspneic, oliguric, with significant abdominal distension, abdominal pain and satisfactory peripheral perfusion. Transferred from the Intermediate Unit to the Intensive Care Center. The notification of suspected dengue was carried out. He presented metabolic acidosis, which was performed volumetric expansion with 1000 ml of saline solution. Evolved with improvement in drowsiness and diuresis. At 00:10, hematocrit of 41.6%, after fluid expansion. At 6:40 am on 4/19/2012, he was found to be very uncomfortable, with reduced breath sounds at the lung bases, and was intubated. He was sedated with midazolam, fentanyl and ketamine. It was punctured by the surgeon for central venous access with placement of a 7 Fr double-lumen catheter. He was evaluated by the nephrologist and continuous hemodialysis was scheduled. Evolved with dehydration, shock, mixed acidosis, hypotransparency in both hemithoraces. Administration of norepinephrine, dobutamine and cefepime was started. Two more water expansions were performed with saline solution. An echocardiogram was performed, which was normal. Concentrate of red blood cells, platelets and vitamin K was prescribed. Blood was collected for serology for dengue, blood culture, hemogram and biochemistry. Chest X-ray with the 4 quadrants involved. Norepinephrine infusion was reduced, because the patient was evolving with a tendency to arterial hypertension. Plasma prescribed. Having a clotting disorder.

At 18:30, the femoral vein was punctured by the surgeon without intercurrences. The radial artery was punctured for better blood pressure control. He remained very serious, in anasarca and acidosis. He evolved with bradycardia, having been performed external cardiac massage, and administration of adrenaline, atropine and bicarbonate. In use of noradrenaline and dobutamine in continuous infusion. Chest drainage was performed bilaterally, with improvement in heart rate, with 970 ml of pleural fluid being drained, with replacement in 2 steps of 500 ml of saline solution. The left femoral vein was punctured for placement of a hemodialysis catheter by the surgeon, without complications. He required a progressive increase in noradrenaline infusion. At 9:20 pm, it was associated with continuous infusion of adrenaline. He had cardiac arrest at 5:50 am, and resuscitation maneuvers were performed with the infusion of an adrenaline bolus, bicarbonate and 25% glucose, without success. Death was confirmed at 7 am on 4/20/2012. His condition can be classified in ICD 10 under the codes: dengue hemorrhagic fever A91, septic shock A41.9, renal failure N19, respiratory failure T96.9 and clotting disorder D68.9 and anasarca R6O.1.


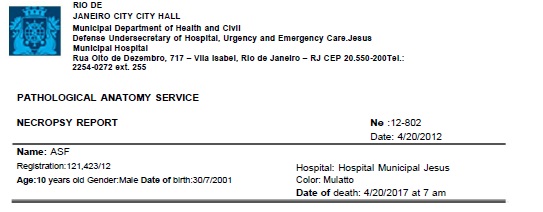


**Cause of Death:** Encephalic Edema

**Main diagnosis:** Hemorrhagic Dengue (Confirmed by positive serology result for dengue IgM antibodies using Dengue IgM kit - Elisa Capture – PanBio)

**Other diagnoses:**

1.- Larynx: Chronic laryngitis with foci of erosion and mucosal necrosis, and hemorrhage in the glottic region, associated with reactive lymphoid follicles. 2.- Trachea: Chronic tracheitis with areas of mucosal necrosis and some reactive lymphoid follicles.

3.- Lungs: Bronchitis and bronchiolitis, foci of alveolar atelectasis, anthracosis and foci of pleuritis. Bilateral pulmonary edema.

4.- Heart: Right ventricle and left ventricle with foci of initial ischemic injury in striated muscle fibers. Focal, septal, left subendocardial hemorrhage associated with swelling (hydropic degeneration) of striated muscle fibers.

5.- Submandibular salivary glands: Nonspecific chronic sialoadenitis, in foci, bilateral.

6.- Tongue: Reactive lymphoid follicular hyperplasia.

7.- Esophagus: Mild chronic esophagitis.

8.- Stomach: Chronic mild superficial gastritis, with lymphoid follicles and some foci of necrosis and hemorrhage in the mucosa, and hemorrhage in the submucosa.

9.- Duodenum: Chronic duodenitis with reactive lymphoid follicular hyperplasia and extensive areas of mucosal necrosis and hemorrhage in the mucosa and submucosa.

10.- Jejunum and ileum: Several small foci, in the mucosa, with degeneration and hemorrhage. Nonspecific reactive lymphoid follicular hyperplasia.

11.- Colon and rectum: Nonspecific reactive lymphoid follicular hyperplasia.

12.- Liver: Steatosis. Hemorrhage and necrosis in zones 3 (perivenular) and 2 (mediozonal) of the hepatic lobules, diffusely, associated with passive sinusoidal hyperemia, secondary to acute ischemia due to circulatory failure due to shock; mild to moderate lymphocytic infiltrate in the portal spaces (reactive hepatitis).

13.- Spleen and accessory spleen: Congestion and foci of hemorrhage in the red pulp.

14.- Lymph nodes: Secondary lymphocytic depletion, associated with apoptotic lymphocytes and hemogocytosis.

15.- Kidneys: Diffuse tubular alterations resulting from hydroelectrolytic disorders.

16.- Testicles and annexes: Hemorrhage in the spermatic cords, bilaterally.

17.- Neck: Hemorrhage in the soft parts on the right, around the Jugular vein and the Carotid artery, as well as in the soft parts adjacent to the thyroid. Right subclavian hemorrhage.

18.- Bone marrow: General cellularity maintained at the expense of immature and mature granulocytic cells and lymphocytes (some in apoptosis), associated with relative hypoplasia of dysplastic megakaryocytes.

19.- CNS: Hypoxic-ischemic neuronal lesion in cerebral cortex, midbrain, pons, medulla and cerebellum.

20.- Anasarca (generalized edema): Skin and subcutaneous tissue, pulmonary edema, bilateral pleural effusions and moderate ascites.

21.- Ectoscopy: Eutrophic. Right cervical and right subclavian vascular puncture marks, and upper and lower limbs (folds of elbows, wrists, dorsum of hands and feet, and bilateral femoral regions) There are two small, open surgical scars, 1 cm each, bilaterally, on the sides of the chest, 7 cm from the armpits, and old cutaneous scars on the right knee and on the right and left legs.

Fernando Colonna Rosman

CRM 52 52023-5
